# Estimates of the population size and dispersal range of *Anopheles arabiensis* in Northern KwaZulu-Natal, South Africa: implications for a planned pilot programme to release sterile male mosquitoes

**DOI:** 10.1186/s13071-021-04674-w

**Published:** 2021-04-19

**Authors:** Maria L. Kaiser, Oliver R. Wood, David Damiens, Basil D. Brooke, Lizette L. Koekemoer, Givemore Munhenga

**Affiliations:** 1 Centre for Emerging Zoonotic & Parasitic Diseases, National Institute for Communicable Diseases of the National Health Laboratory Service, Johannesburg, South Africa; 2 Wits Research Institute for Malaria, School of Pathology, Faculty of Health Sciences, University of the Witwatersrand, Johannesburg, South Africa; 3 UMR IRD 224, Maladies Infectieuses et Vecteurs-Ecologie-Génétique, Evolution et Contrôle (MIVEGEC), Institut de Recherche Pour Le Développement (IRD) CNRS 5290–Université de Montpellier, Montpellier, France; 4 IRD Réunion/GIP CYROI (Recherche Santé Bio-innovation), Sainte Clotilde, Reunion Island France

**Keywords:** Malaria vector control, Malaria elimination, Male mosquitoes, Population size, Over-flooding ratio, Sterile insect technique

## Abstract

**Background:**

*Anopheles arabiensis* is a major malaria vector, recently implicated as contributing to ongoing residual malaria transmission in South Africa, which feeds and rests both indoors and outdoors. This species is, therefore, not effectively targeted using core malaria vector control interventions alone. Additionally, increasing resistance to available insecticides necessitates investigations into complementary non-insecticide-based vector control methods for outdoor-resting mosquitoes. The feasibility of the sterile insect technique (SIT) as a complementary vector control intervention is being investigated in South Africa. Successful implementation of an SIT programme largely depends on inundating a target insect population with sterilized laboratory-bred males. Therefore, knowledge of the native population size and dispersal ability of released sterile laboratory-reared males is critical. In this study, we estimated the male *An. arabiensis* population size and the dispersal of released males in an area targeted for a pilot sterile male release programme.

**Methods:**

Three separate releases were performed within a 2-year period. Approximately 5000–15,000 laboratory-reared male *An. arabiensis* (KWAG) were produced and marked for mark–release–recapture experiments. To recapture released mosquitoes, cloth tubes were deployed in widening concentric circles. The average dispersal distance of released males was calculated and the wild male *An. arabiensis* population size was estimated using two Lincoln index formulae. The natural population was sampled concurrently and *Anopheles* species diversity examined.

**Results:**

The *Anopheles gambiae* complex and *An. funestus* group species made up the majority of wild collections along with other anophelines. The *An. arabiensis* population size was estimated to be between 550 and 9500 males per hectare depending on time of year, weather conditions and method used. Average dispersal distance of marked males ranged from 58 to 86 m. Marked males were found in swarms with wild males, indicating that laboratory-reared males are able to locate and participate in mating swarms.

**Conclusions:**

It was logistically feasible to conduct mark–release–recapture studies at the current scale. The population size estimates obtained may provide a guideline for the initial number of males to use for a pending SIT pilot trial. It is promising for future SIT trials that laboratory-reared marked males participated in natural swarms, appearing at the right place at the right time.

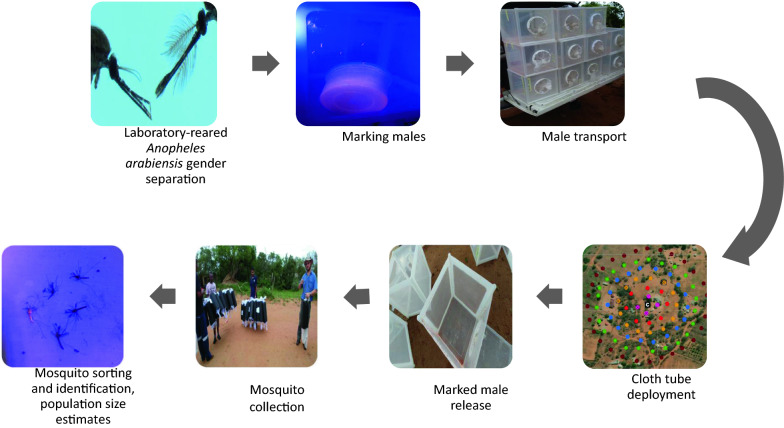

**Supplementary Information:**

The online version contains supplementary material available at 10.1186/s13071-021-04674-w.

## Background

South Africa’s malarious areas include the northeastern border regions of three provinces: KwaZulu-Natal (KZN), Mpumalanga and Limpopo [1]. These provinces border Eswatini (formally Swaziland), Mozambique, Zimbabwe and Botswana. Of these countries, Mozambique is an especially high-risk area for malaria [1] and poses challenges for malaria elimination initiatives in South Africa. In KZN, the highest malaria incidence occurs in the northeastern part of the province, particularly near the border with Mozambique [2].

The major malaria vectors in South Africa are *Anopheles funestus* and *An. arabiensis* [3], with the former now nearly eliminated by the provincial indoor residual spraying (IRS) programmes and the latter recently implicated in ongoing residual transmission [4]. Potential secondary vectors include *An. merus*, *An. coustani*, *An. marshallii*, *An. pharoesnis*,* An. rivulorum* and *An. leesoni* [5]. *Anopheles vaneedeni* and *An. parensis* have recently been implicated as secondary vectors in South Africa [6, 7].

In the almost complete absence of *An. funestus*, *An. arabiensis*, a species that displays variable feeding and resting behaviour, is currently implicated as the major vector in the ongoing residual malaria transmission in South Africa [4]. *Anopheles arabiensis* will feed off animals (predominantly cattle) as well as humans and may rest outdoors as well as indoors [8–10]. This poses a problem for South Africa’s malaria elimination agenda [11] because the provincial malaria control programmes rely heavily on IRS and, to a lesser extent, larval source management, for vector control [3, 11, 12]. Outdoor-resting vector populations are ineffectively targeted by IRS, requiring the adoption of additional methods for their control.

Additional vector control tools that can target outdoor-feeding and resting vectors and are not insecticide based are urgently required. Environmentally friendly and species-specific, the sterile insect technique (SIT) is one such tool under consideration. SIT, a concept conceived by Knipling in the 1930s [13], has previously been considered for use against malaria vector mosquitoes, but was abandoned due to various factors, including public perceptions and political instability [14]. The technique has been used successfully against several insect pests, including the New World screwworm fly [15], pink bollworm [16], fruit flies [17], tsetse fly [18] and codling moth [19]. SIT involves the mass production and release of sterilized laboratory-reared male insects that have retained sufficient fitness to compete with wild males for wild females. Inundative releases of sterile males have been shown to induce sufficient sterility in wild females to effectively suppress, and in some instances eliminate, targeted insect populations [15–19].

A project to assess the feasibility of using the SIT for the control of *An. arabiensis* in South Africa has been underway since 2011 [20]. A suitable field site for pilot field releases has been established at Mamfene, northern KwaZulu-Natal Province [4]. Regular vector surveillance and community engagement has been ongoing at this site since 2014 [4, 21], providing baseline data for annual and seasonal fluctuations in malaria vector density and composition. An *An. arabiensis* genetic sexing strain has been developed by introgressing a genetic sexing mechanism into the genetic background of the target population at Mamfene [22, 23]. The optimal irradiation dose for sterilizing males has been established, and the competitiveness of wild* versus* laboratory-reared sterilized males determined [23, 24].

The Mamfene field site is well suited for an SIT intervention as the malaria incidence in KwaZulu-Natal is currently less than one case per 1000 residents [11]. The site is located in a malaria hotspot where annual vector control using IRS is conducted (National Department of Health). The surrounding areas are not sprayed unless a malaria case is detected, in which case spraying is conducted using foci clearing protocols [11].

Before a pilot SIT trial can be implemented, it is necessary to estimate the *An. arabiensis* population size at the target site in order to be able to estimate the number of sterilized males required to inundate the target population at the required ratio (over-flooding ratio). One of the most common methods used to estimate population size is the mark–release–recapture technique (MRR) [25]. Using MRR, population size can be estimated by releasing a known number of marked individuals into the target area. Individuals of that species are subsequently captured and, based on the proportion of marked individuals captured compared to wild (unmarked) individuals captured, an estimate of the population size can be calculated. MRR can also be used to measure dispersal [25, 26]. Many MRR studies on mosquitoes have been conducted [25–30], mostly on females. However, some recent studies have focused on males [27, 28, 30].

The aim of this study was to estimate the *An. arabiensis* population size at the Mamfene intervention site, as well as gain insight into dispersal ability of laboratory-reared males. In addition, the logistics and feasibility of producing laboratory-reared males in Johannesburg and then transporting and releasing them in Mamfene, KwaZulu-Natal Province were determined.

## Methods

### Field experimental site

**Fig. 1:**
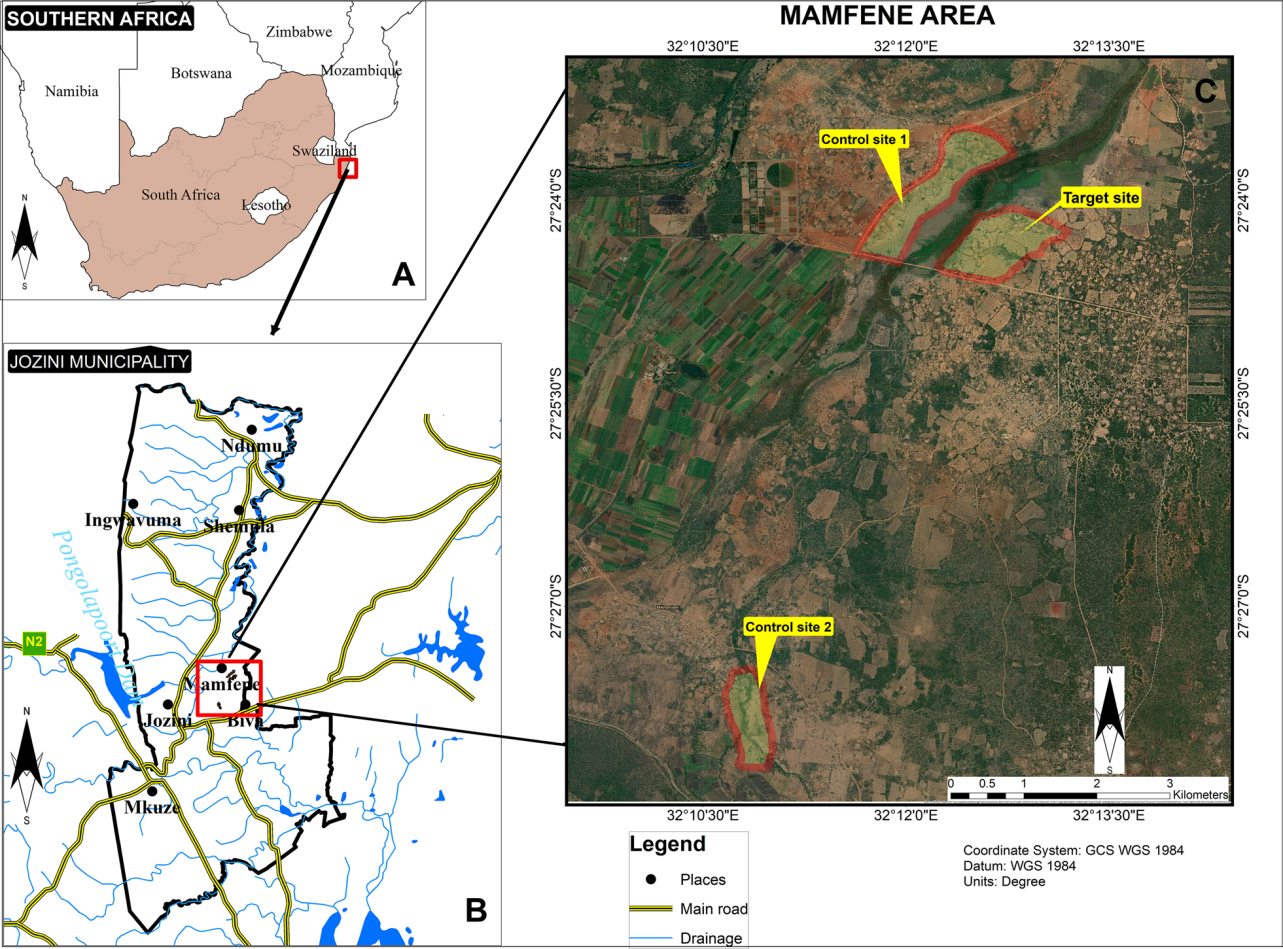
**a** Location of the study site in KwaZulu-Natal Province, South Africa. **b** View of the Jozini Municipality, showing the location of Mamfene. **c** Study site showing the target site and the two control sites. Control site one is adjacent to the target site and control site two is approximately 8 km away. The area of each section is: target site, 0.48 km^2^; control site one, 0.70 km^2^; control site two, 0.26 km^2^

All field activities were conducted at Mamfene in the uMkhanyakude district of KwaZulu-Natal Province (Fig. 1a). The experimental area is divided into three sites, two control sites and one target or intervention site (Fig. 1b). Mamfene is predominantly rural with subsistence farming at the household level, and most of the surrounding areas are occupied by sugarcane plantations. The local community is predominantly housed in small westernized buildings with a few isolated traditional mud structures present. Cattle and goats are kept by some members of the community and many of them are allowed to graze freely in the fields adjacent to the households, and along the roads. During the rainy season, puddles are formed, creating potential oviposition sites for gravid *An. arabiensis* which tend to oviposit in small temporary water bodies dependent on rainfall [10, 31]. There is a marshy area located between control site one and the target site that also provides suitable mosquito larval sites. A stream runs along the southern side of control site two. Rainfall is highest from September to April, coinciding with the malaria season. Average temperature is 22.3 °C, with June being the coolest and February the hottest month, respectively. Average rainfall is 569 mm per year [32].

### Biological material

The mosquitoes were drawn from KWAG, an *An. arabiensis* colony that originated from Mamfene, KwaZulu-Natal Province and established in 2005 [33]. This colony has been reared through many generations in the laboratory since its establishment; however, generation numbers were not recorded. KWAG is maintained in the Hugh Patterson insectary at the National Institute for Communicable Diseases (NICD) in Johannesburg.

### Production of KWAG males for release

The Hugh Patterson insectary has two rearing rooms. The room reserved for KWAG larval stages was maintained at (mean ± standard deviation [SD]) 32 ± 3 °C and 80 ± 5% relative humidity (RH) to reduce larval developmental time. Larvae were fed a mixture of finely ground dog biscuits (Beeno^®^, Kempton Park, South Africa) and brewer’s yeast (Vital^®^; Vital Health Foods, Cape Town, South Africa) [34]. A second room was used to house the adults and was maintained at the standard (25 °C) rearing temperature [34]. All adult mosquitoes were fed 10% sugar solution* ad libitum* and adult females were offered blood meals at least twice per week using an artificial membrane feeding system (Hemotek^®^; Hemotek Ltd., Blackburn, UK).

To obtain males for MRR, pupae were separated from larvae in batches using the vortex induction technique described in the International Atomic Energy Agency (IAEA) guidelines [35]. The number of pupae collected was established using volumetric estimation in batches of no greater than 5000 pupae [35]. After separation from larvae, pupae were placed into BugDorm^®^ cages (BugDorm-1: 30 × 30 × 30 cm; BugDorm, MegaView Science Co., Ltd, Taichung, Taiwan; hereafter referred to as “cage”) in batches of 1500 pupae per cage, provided with 10% sugar solution and allowed to emerge overnight. One to two days after adult emergence males were separated from females based on antennal morphology and transferred into new cages at a density of 1000 males per cage. These were maintained on 10% sugar solution* ad libitum* until marking.

### Marking of adult males prior to release

Males were marked with fluorescent dust (Day Glo series; Day-Glo Color Corp., Cleveland, OH, USA) 2 to 3 days prior to release. The following dust colours were used: pink, yellow and orange (see Additional file 1: Table S1). Prior to marking, the sugar water was removed from each cage, and the number of dead males was recorded and the dead males removed. The mosquitoes were immobilized by placing each cage into a refrigerator for 2–5 min. Immobilized mosquitoes were tipped into a pre-dusted plastic container using a large funnel. Plastic containers (1 l) were prepared for dusting 1000 males by adding 0.05 g of fluorescent dust, weighed using an analytical balance (model Kern ABT 120-5DM, United Scientific Equipment Pte Ltd, Singapore; model Adam PW124, Labex, 88 17th Avenue, Edenvale, Johannesburg, 1609) according to the methods developed by Culbert et al. [36], but modified for larger numbers. Any escaped mosquitoes or those not successfully transferred into the dusting container were counted, recorded and excluded from the calculation of final male release number. The 10% sugar solution was provided* ad libitum *immediately following marking. This method successfully marked all of the males. In a similar separate experiment, no significant effect on longevity on marked* versus* unmarked males was observed [36]. For the mark–release–recapture experiments with more than one release location, two dust colours were used, one for each site.

### Cloth tube construction

**Fig. 2:**
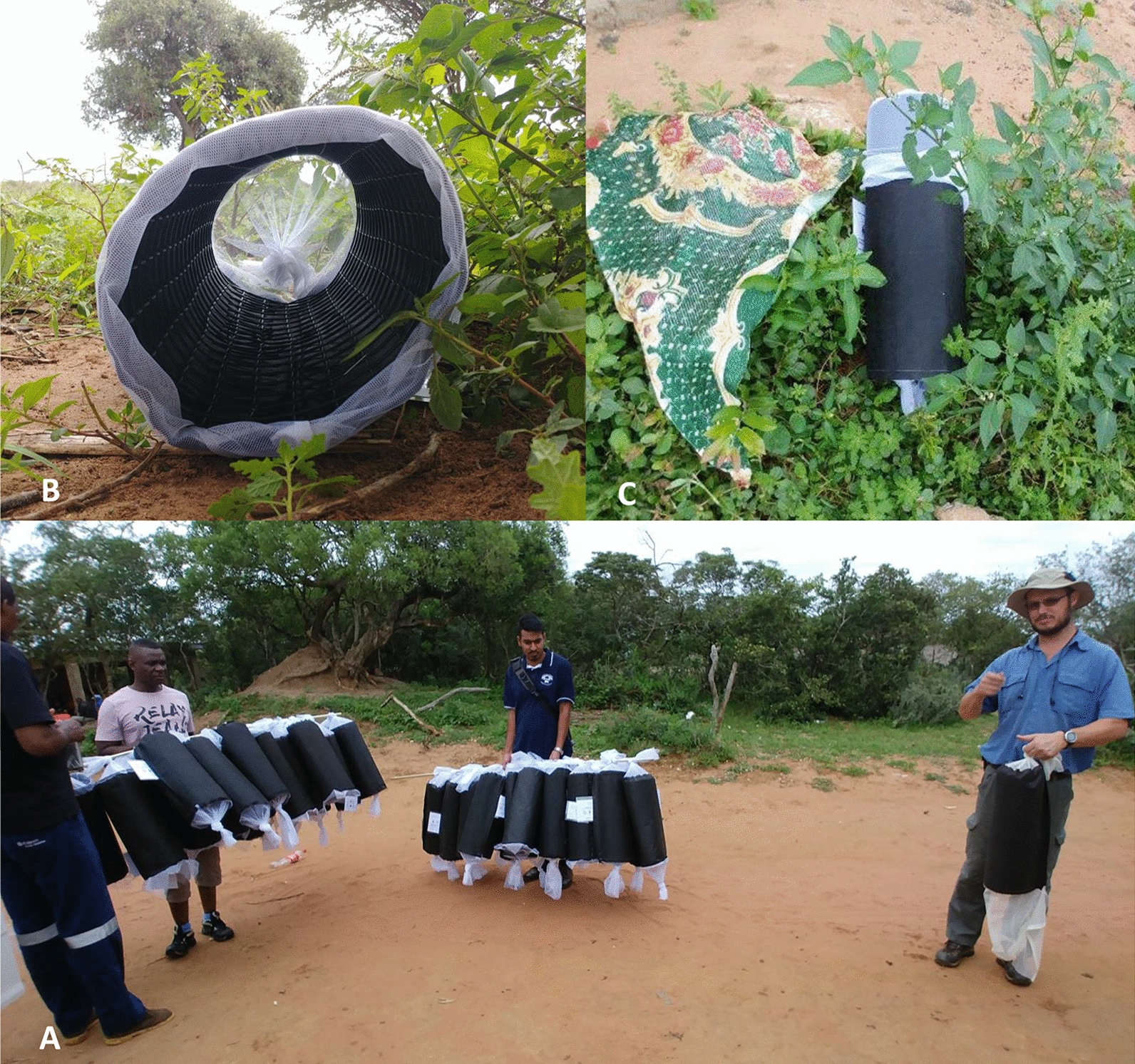
**a** Cloth tubes used during mark–release–recapture experiments strung onto a pole for collection and deployment of multiple tubes at once. **b**, **c** Typical placement in the field. Note the netting sleeves (one closed and one open) used to close the tube for more rapid collection

Previously designed sentinel style resting sites, named cloth tubes (Fig. 2), were used for collections [24]. Cloth tubes with a diameter of 18–20 cm and length of 50–54 cm were constructed using a tubular frame made from plastic garden mesh fencing, a black fabric skin and a pair of netting sleeves capping each end. A couple of hand-stitched tacks between the frame and fabric were used to keep the frame and skin together. Laminated labels were stapled to the hem for trap identification, and nails were driven through the hem to anchor the traps to the ground when deployed. The cloth tubes for the pilot trial were capped with a net at one end, with the other end left open; however, the design was subsequently improved, as described above, so that it was possible to close both ends during collection. This latter design prevented the escape of any mosquitoes inside and also aided in distributing and collecting more tubes at once (Fig. 2a). Cloth tubes were placed either horizontally or at an angle of approximately 45° to the ground when deployed (Fig. 2b, c).

### Transport of males from the insectary to the field laboratory and release sites

The NICD insectary is located approximately 500 km from the field site by road. Marked males were transported in BugDorm-1^®^ cages placed in the enclosed canopy at the back of a field vehicle (pick-up truck), at ambient temperature with access to 10% sugar solution. To maintain humidity and a cooler temperature during transportation, damp towels were draped over the cages. This method is used routinely by the laboratory to maintain mosquitoes in good condition during transport. On arrival at the field laboratory, marked males were left inside the transport vehicles overnight. These males were provided with fresh 10% sugar solution the following morning and moved into the field laboratory until release. During this holding phase, all cages were visually scanned for females aided by a volunteer who placed an arm onto the mesh side of the cage. Any females found were recorded and removed. The number of females found was generally low, ≤ 5 per cage (0.5%), with some cages free of females. Males were transported in their cages to the release sites in the field vehicles. For mark–release–recapture one (see following section), with two release sites, two field vehicles were used, one for each release site (and therefore one vehicle for each dust colour).

### Release location selection and the setting up of cloth tubes in the field

Three releases, referred to as mark–release–recapture pilot trial (MRR^PT^), the mark–release–recapture one (MRRI) experiment and the mark–release–recapture two (MRRII) experiment, were conducted between November 2016 and October 2018 (Additional file 1: Table S1).

**Fig. 3:**
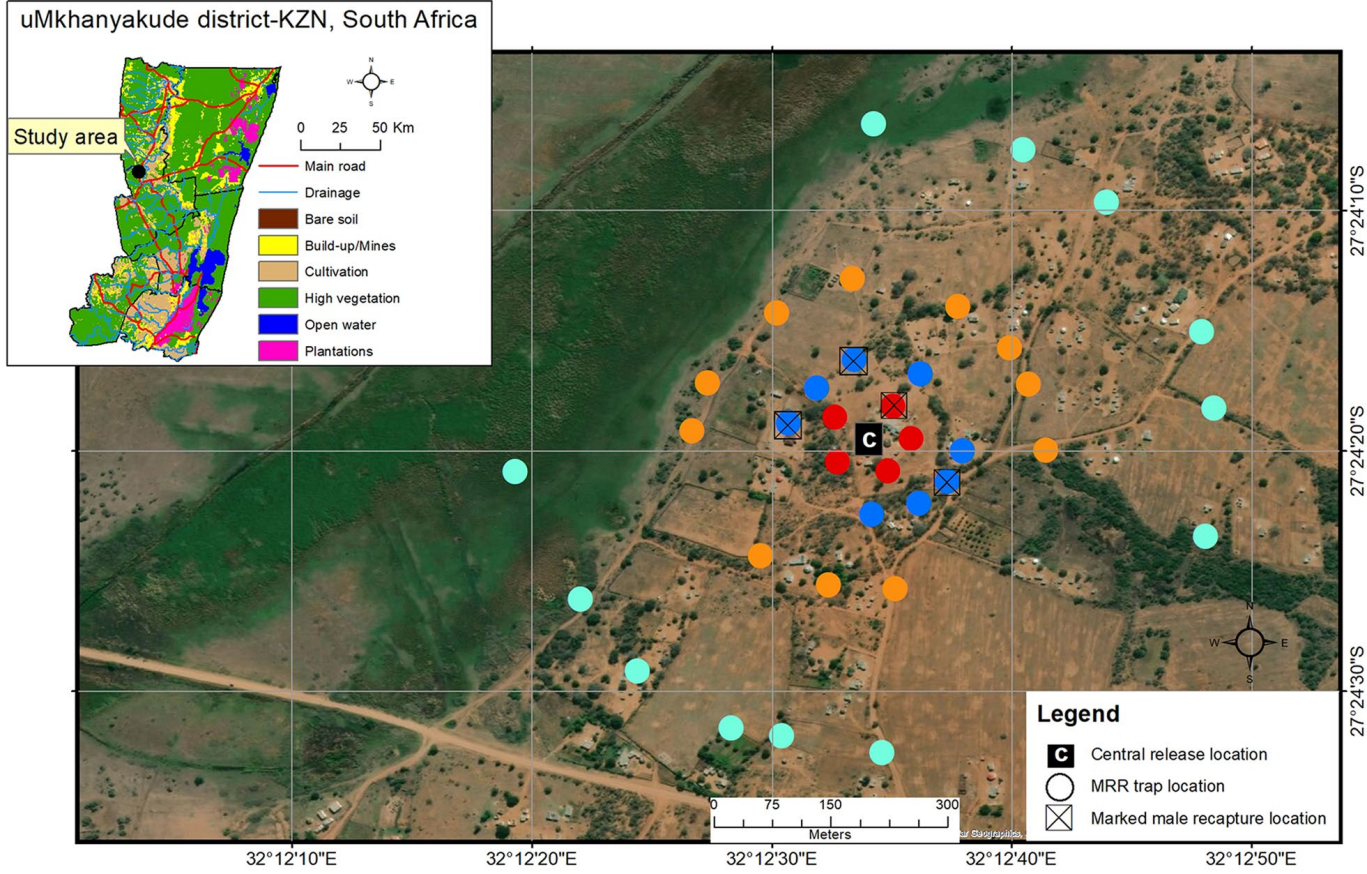
Cloth tube set-up around the central release site (*C*) in the mark–release–recapture (*MRR*) pilot trial (MRR^PT^). Cloth tubes were placed in concentric circles around C at radii of 50 m (red), 100 m (blue), 200 m (orange) and 400 m (pale green). Insert shows the land cover type in the study area

**Fig. 4:**
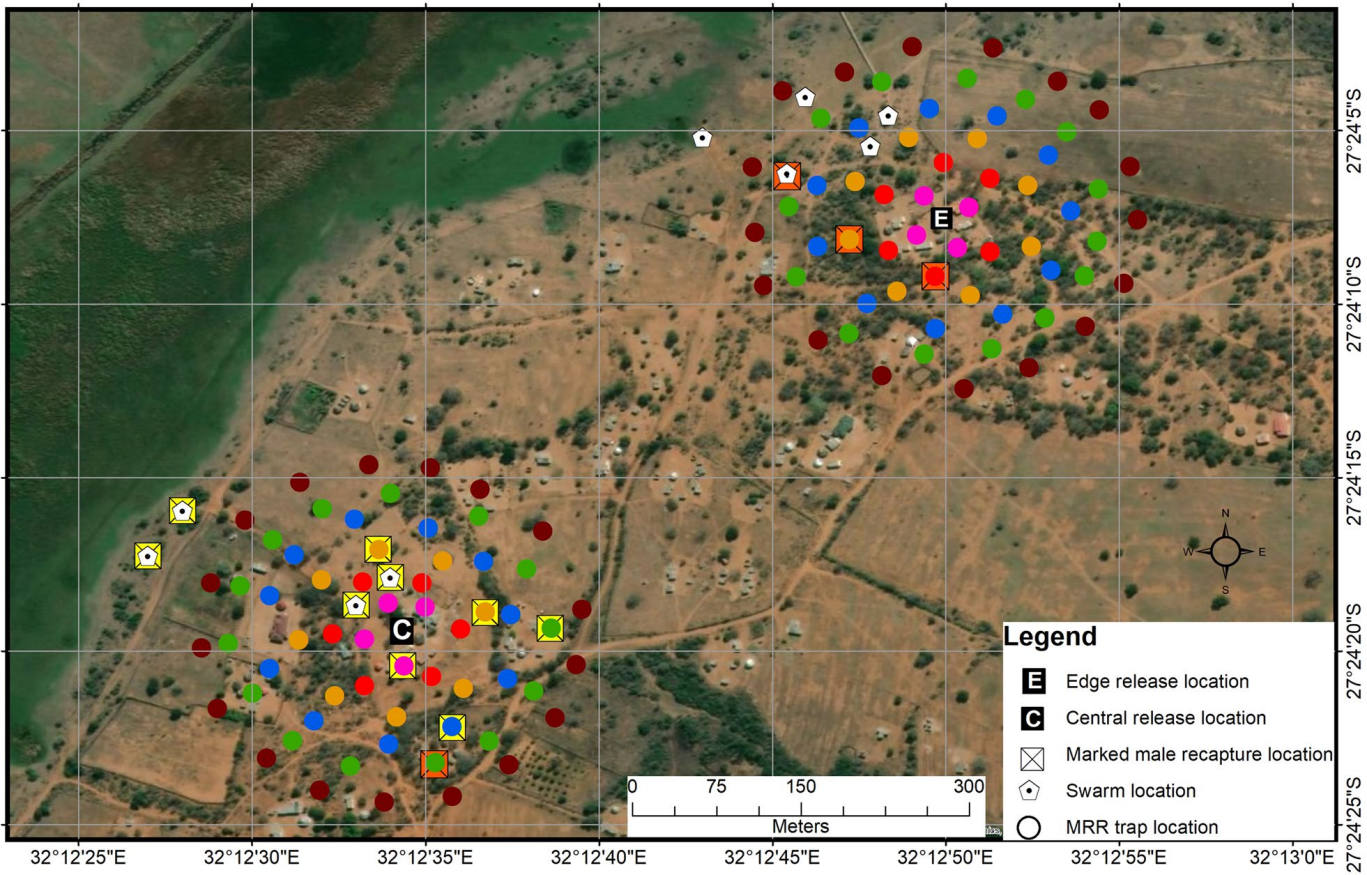
Distribution of cloth tubes in the mark–release–recapture (*MRR*) one (MRRI) experiment. Cloth tubes were placed in concentric circles around the central (*C*) and edge (*E*) release points at radii of 25 m (pink), 50 m (red), 75 m (beige), 100 m (blue),125 m (green) and 150 m (burgundy). Swarm locations are also indicated. Swarms and tubes with marked males are indicated with the colour of marked males (yellow released from C and orange released from E)

**Fig. 5:**
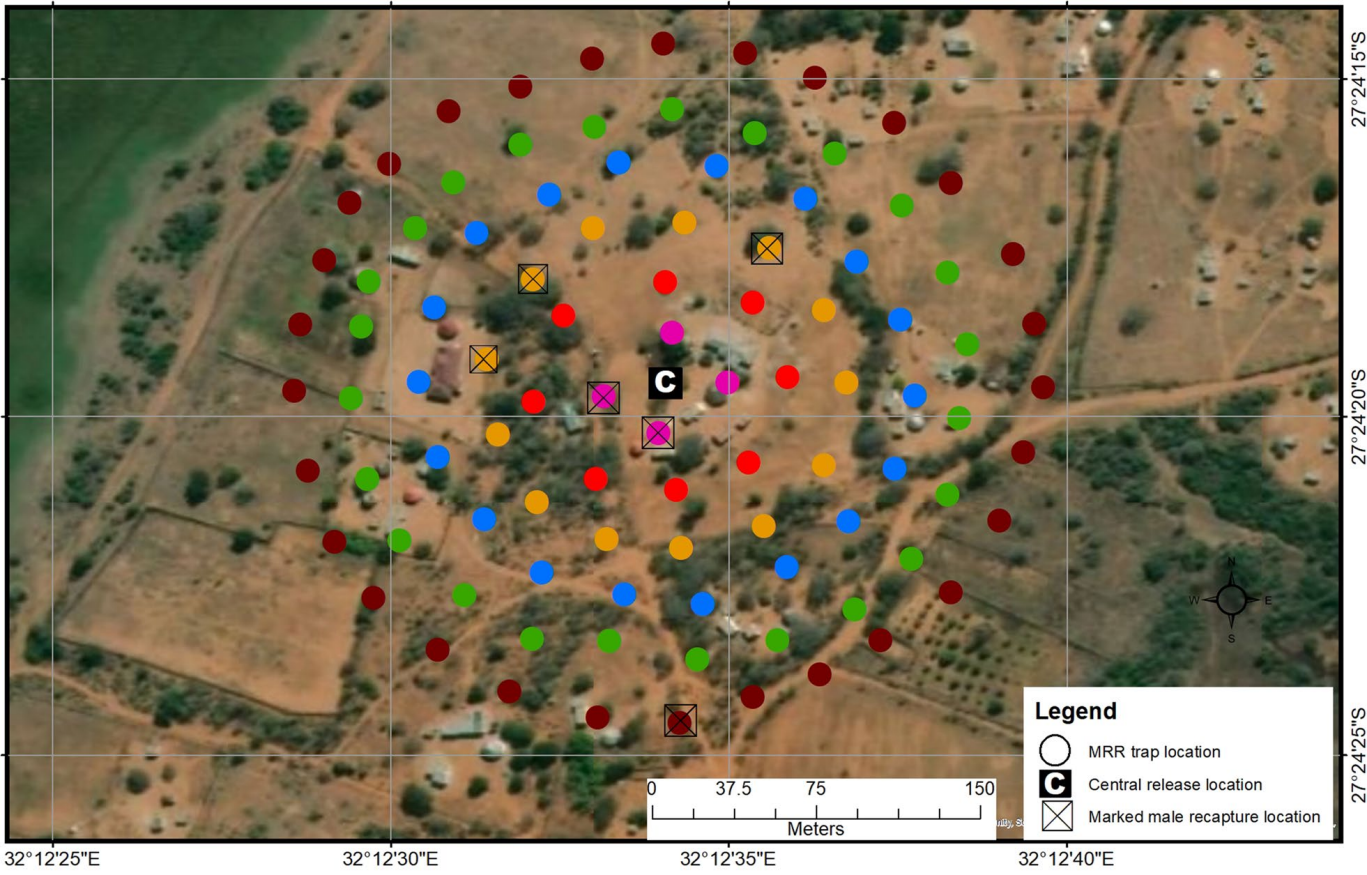
Distribution of cloth tubes in the mark–release–recapture (*MMR*) two (MRRII) experiment. Cloth tubes were placed in concentric circles around the central (*C*) release point at radii of 25 m (pink), 50 m (red), 75 m (beige), 100 m (blue), 125 m (green) and 150 m (burgundy). Tubes with marked males are indicated

The release site (household) used in MRR^PT^, MRRI and MRRII was selected based on its central position in the target site (Figs. 3–5). This site was named C (centre), with co-ordinates 27°24′19.68″S; 32°12′34.37″E. For MRRI, an additional release point at the edge of the target site, approximately 550 m from the central release site, was selected and named E (edge), with co-ordinates 27°24′7.70″S; 32°12′49.64″E (Fig. 4). Prior to setting up the tubes, permission was sought from household owners. The density of the cloth tubes deployed to the field increased with each release in an attempt to improve the chances of capturing and recapturing mosquitoes (Table 1). In MRR^PT^, the cloth tubes were placed at a maximum distance of 400 m (Fig. 3; Table 1) from the release site; however, in subsequent experiments they were placed up to a maximum of 150 m from the release site as marked males were only captured in tubes up to 100 m from the release site during MRR^PT^ (Figs. 4, 5; Table 1). The position of each cloth tube was mapped using a hand-held GPS device (Garmin eTrex 20×; Garmin Ltd., Schaffhausen, Switzerland) (Figs. 3–5).

**Table 1:** Number of cloth tubes by distance from release site for mark–release–recapture pilot trial and mark–release–recapture one and two experiments

Distance of cloth tube from the release point (m)	MRR experiment (release location) and number of cloth tubes per radius		
	MRR^PT^ (C)	MRRI (C and E)	MRRII (C)
25	–	4	4
50	5	6	8
75	–	8	13
100	8	11	18
125	–	14	24
150	–	17^a^	29
200	11	–	–
400	12	–	–
Total cloth tubes	36	60^a^	96

C, Central release point; E, edge release point; MRR^PT^, mark–release–recapture pilot trial; MRRI, MRRII, mark–release–recapture one and two experiments, respectively

^a^For MRRI, E had an additional trap at 150 m (two traps were mistakenly labelled 150.14 so one became 150.14B), bringing the total number of traps at E to 61

### Male releases

The males were transported (as described previously) from the field laboratory to the release locations in BugDorm-1 cages. The duration of the trip from the field laboratory to release sites is approximately 25 min, and arrival at the release locations was timed to be at least 30 min before the planned releases. All releases were conducted outdoors between 10 and 20 min before sunset using 2- to 5-day-old marked males.

To release males, the cage tops were removed and the cages tapped to encourage the marked males to fly out. Any dead males, or those incapable of flying out of the cages, were retained. Cages were re-closed after releases and the remaining males later counted to calculate the actual number of males released. In the MRRI experiment, males were simultaneously released at two sites (Fig. 4) by two independent release teams.

### Mosquito collection from cloth tubes

All collections occurred daily, beginning at dawn just as it became light, and were complete within 1–2 h. During MRR^PT^, mosquitoes were collected directly from the cloth tubes (which were open at one end and capped at the other end) in the field by illuminating the trap using a torch light and collecting any mosquitoes using a manual aspirator. Collected mosquitoes were immediately placed into polystyrene cups labelled with the tube name and date of collection, and subsequently transported to the field laboratory for morphological species identification using standard keys [8, 9]. During subsequent releases (MRRI and II), entire cloth tubes were collected and replaced straightaway with a new ones labelled as previously described. Cloth tubes were placed into the field with one sleeve closed and the other end open to allow mosquito entry. Upon collection, the open sleeve was closed for transport to the laboratory.

### Swarm searches

Daily swarm searches were performed each evening following releases. Searches started approximately half an hour before sunset and ended when it became too dark to see. Swarms were detected by observing the horizon towards the sun in potential swarming areas, as described in Diabaté et al. [37]. Potential swarming sites were identified above small bushes, walking path intersections, above mounds or depressions in the ground and near the leafy border of tall trees. Such contrasting areas are considered to be potential swarm markers [38, 39]. Samples were collected from identified swarms (Fig. 4) using a sweep net (except directly after releases). Sweep nets were constructed specifically for collecting swarms using a plastic hula-hoop (diameter:  approx. 70 cm) fitted with a large conical net (length: 1.5 m) and a square lashed to a notched handle (length: 1.8–2 m) made of thick bamboo (Additional file 2). Swarm catches were immediately inspected (in sweep nets) by torch light for the presence of mosquitoes. If mosquitoes were present, these were transferred to polystyrene cups using an aspirator and kept for morphological identification [8, 9] in the field laboratory the following day. The date, time, swarm height, swarm size and location of each swarm were recorded.

### Mosquito identification and processing in the laboratory

All collected mosquito specimens were transported to the field laboratory daily for initial processing. Each cloth tube taken to the laboratory was checked for the presence of mosquitoes by looking through one end of the tube towards the light while gently tapping and rotating the tube. If mosquitoes were found, these were carefully aspirated out. The cloth tubes were then fully opened onto a sheet of white paper to recover any dead mosquitoes. All anopheline mosquitoes found in each tube were placed into disposable cups and appropriately labelled with the tube name and collection date. Anopheline mosquitoes collected alive were anaesthetised using ethyl acetate or dry ice before morphological identification [8, 9]. All mosquitoes were scanned for fluorescent dust markings under the microscope with the aid of a UV torch (9 LED UV Scorpion flashlight; Zartek, Johannesburg, South Africa), although markings were readily visible by eye. After this initial processing, all specimens were stored in individual silica tubes and transported to Johannesburg by road at the completion of the field work for further analyses. Individual specimen records were maintained on tube identification, date, species (by morphology) and whether males captured were marked (including colour) or unmarked (wild males). Once in the Johannesburg laboratory, *An. gambiae* complex and *An. funestus* group specimens were identified to species using the PCR-based methods of Scott et al. [40] and Koekemoer et al. [41], respectively.

### Dispersal distance

The average dispersal of released males for each experiment was calculated by adding the distance of each trap (from the release point) in which each marked male was collected and then dividing this by the total number of males recaptured for that experiment. Maximum dispersal distance of marked males was also determined.

### Population size estimation and calculation of daily survival probability and average life expectancy

The number of *An. arabiensis* males was estimated using two modified Lincoln indexes for low recapture rates. Both indices were used to give a minimum and a maximum number for release. This was done out of concern about releasing too few sterile males as this would reduce the efficacy of the potential SIT trial.

The first index takes into account daily survival rate ([42] as referenced in [43]) according to the following equation: $P=[a{s}^{t}\left(n-r+1\right)/ (r+1)$, where *P* is the estimated population size, *a* is the number of marked males released, *s* is the estimated probability of daily survival, *t* is the sampling day post release, *n* is the total number of marked and unmarked males captured and *r* is the number of marked males recaptured ([42], as referenced in [43]). Daily survival probability (*s*  in equation) is calculated by the regression of the total number of marked males transformed by $\mathrm{log} (x+1)$ in all traps on the day post release. This was calculated for 5 days after release. Survival probability was estimated using Statistix version 8.0 (Analytical Software, Tallahassee, FL, USA) from the results of the antilog of the slope of the regression line [44, 45]. Average life expectancy was calculated by the formula: $1/-\mathrm{ln}(s)$ (see [46]).

The second index is the standard Lincoln index used to estimate populations from MRR experiments when *r*, the number of recaptured individuals, is < 20 [43]. This index assumes constant daily survival and is described by the equation: $P=\frac{a\left(n+1\right)}{r+1}$

, where *a* is the number of samples originally marked, *n* is the total number of recaptures and *r* is the number of recaptured marked individuals [43].

The estimated number of males is expressed as the total population estimate and as the number of males per hectare.

### Weather data

All weather data were obtained from the Makhathini Research Station (27°23′42.45″S; 32°10′48.48″E), located approximately 3.2 km from release location C. Statistical analyses were performed using Statistix version 8.0. Mean temperature, relative humidity and mean daily wind speed on the day of release and subsequent mosquito collection days were calculated. Mean temperature and relative humidity for the release and collection periods were compared for each experiment using one-way analysis of variance (ANOVA); if significant, a Bonferroni post-hoc test was conducted to separate the means. Average wind direction and wind speed were also determined for each release. In addition, the rainfall pattern was investigated for the 4-week period prior to MRRII.

## Results

### Numbers released, percentage recapture, survival probability, average life expectancy and population size estimation

The number of marked males released, the number recaptured, the daily survival probability and the estimated population size for each experiment are given in Table 2. The population sizes for the two release locations used in MRRI were calculated separately. The largest population size estimate was obtained during MRRI at E and the smallest was obtained during MRRII at C. The percentage recapture of marked males from deployed cloth tubes was 0.09, 0.11, 0.06 and 0.04% for MRR^PT^, MRRI at C, MRRI at E and MRRII, respectively. The average life expectancy for each experiment, using the data from cloth tubes only (including the 1 clay pot used in MRR^PT^), was 2.94 days for MRR^PT^, 2.59 days for MRRI C, 4.48 days for MRRI E and 2.59 days for MRRII. The average *An. arabiensis* male population sizes, as calculated for the first 2 days of collections, were 539 (MRR^PT^), 783 (MRRI at C), 6213 (MRRI at E) and 401 (MRRII) males per hectare using the index that takes daily survival into account [42]. Using the simple index [43], the population size averages for the first 2 days after releases were: 2819 (MRR^PT^) 1936 (MRRI at C), 9643 (MRRI at E) and 2193 (MRRII) males per hectare (see Table 2 for more details).

**Table 2:** Estimated total and per hectare population size of *Anopheles arabiensis* males in Mamfene, KwaZulu-Natal, South Africa

Experiment/Day	Number released (day 0)	Number of wild males	Number of marked males		Survival probability per day (s^t^ )^a^	Estimate 1 (per ha)^b^	Estimate 2 (per ha)^b^
MRR^PT^ C/1	7871	0	3		0.71	1397 (445)	7871 (2505)
MRR^PT^ C/2		1	3		0.5	1984 (632)	9839 (3132)
MRR^PT^ C/3		0	0		0.36	2817 (897)	7871 (2505)
MRR^PT^ C/4		0	1		0.25	1000 (318)	7871 (2505)
MRR^PT^ C/5		2	0		0.18	4260 (1 356)	23,613 (7516)
MRRI C/1	5262	6	4		0.68	5009 (709)	11,576 (1638)
MRRI C/2		4	1		0.46	6051 (856)	15,786 (2233)
MRRI C/3		4	1		0.31	4078 (577)	15,786 (2233)
MRRI C/4		4	0		0.21	5525 (782)	26,310 (3722)
MRRI C/5		2	1^c^		0.15	2368 (335)	15,786 (2233)
MRRI E/1	5453	9	2		0.8	14,541 (2057)	21,812 (3086)
MRRI E/2		20	0		0.64	73,288 (10,368)	114,513 (16,200)
MRRI E/3		30	1		0.51	43,275 (6122)	87,248 (12,343)
MRRI E/4		8	0		0.41	20,102 (2844)	49,077 (6943)
MRRI E/5		13	0		0.33	25,040 (3542)	76,342 (10,800)
MRRII C/1	~ 15,500	0	4		0.68	2108 (298)	15,500 (2193)
MRRII C/2		0	1		0.46	3565 (504)	15,500 (2193)
MRRII C/3		0	1		0.31	2403 (340)	15,500 (2193)
MRRII C/4		0	0		0.21	3255 (460)	15,500 (2193)
MRRII C/5		1	0		0.15	4650 (658)	31,000 (4386)

^a^Survival probability as calculated for estimate 1

^b^Estimate 1 was obtained using the procedure of [42], and Estimate 2 using [43]

^c^An orange marked male was captured at C which had travelled over 600 m to reach the trap at E. This male was excluded from the population size estimation because it was beyond the area used for the calculation, reducing the total captured to 26 and the marked recaptures to 6 for C during MRRI

### Swarms

Released males were observed to form their own swarms on the day of release near the release location during MRR^PT^ and MRR II. Figure 4 indicates swarms that were found during MRRI. Of these, 55.55% (*n* = 5 swarms) contained marked males (Table 3). Notably, a single marked male was recaptured 8 days after release while checking for swarms during routine surveillance work (Table 3). Although swarms were found during MRRII, these were *An. marshallii* complex with a few *An. coustani*. Table 4 shows the number of marked males released per site in MRRI and the number of *An. arabiensis* males captured in swarms. Due to the limited amount of data obtained from swarms in this series of experiments, no population size estimates were made from the swarms.

**Table 3:** *Anopheles* male mosquitoes captured in swarms (ordered by day post release) at the central and edge locations during MRRI in Mamfene, Jozini, KwaZulu-Natal Province, South Africa

Day	Location (GPS co-ordinates)	Marker	*Anopheles* species present	Number of marked to wild *An. arabiensis* males
1	1+ km from E (27°23′52.16ʺS; 32°13′24.84 E)	Ground depression	*An. arabiensis*	0:12
2	150 m from E in Marsh (27°24′4.38ʺS; 32°12′45.55ʺE)	Vegetation on edge of marshy area	Other species	NA
2	200 m from E in Marsh (27°24′5.19ʺS; 32°12′42.59ʺE)	Mound in marsh	*An. pharoensis*	NA
2	125 m from E (27°24′6.31ʺS; 32°12′45.66ʺE)	Mound with log	*An. arabiensis*	1or:4
3	125 m from E (27°24′6.31ʺS; 32°12′45.66ʺE)	Mound with log	*An. pharoensis*, *An arabiensis*	0:1
3	95 m from E (27°24′5.01ʺS; 32°12′47.88ʺE)	Small bush	*An. arabiensis*	0:14
3	55 m from C (27°24′18.68ʺS; 32°12′32.88ʺE)	Bare ground	*An. arabiensis*	1ye:1
3	60 m from C (27°24′17.87ʺS; 32°12′33.84ʺE)	Between two bushes (~ 9 m to each bush)	*An. arabiensis*	3ye:9
3	215 m from C (27°24′15.95ʺS; 32°12′27.89ʺE)	Clearing between two bushes	*An. arabiensis*, *An. parensis*	1ye:9
4	95 m from E (27°24′5.01ʺS; 32°12′47.88ʺE)	Small bush	*An. arabiensis*	0:2
8	210 m from C (27°24′17.26ʺS; 32°12′27.35ʺE)	Large bush near CP 9B	*An. arabiensis*	1ye:0

CP, Clay pot; NA, not applicable (no *An. arabiensis* in swarm); or, orange marked male; ye, yellow marked male

**Table 4:** Total *Anopheles arabiensis* males and marked released males captured from swarms only during MRRI, Mamfene, Jozini, KwaZulu-Natal Province, South Africa

Release point	Number of marked males released	Total *An. arabiensis* males in swarms	Total marked males recaptured (swarms)
C	5262	24	5
E	5453	22	1

### Species composition, distribution and dispersal

#### Pilot trial (MRR^PT^)

During MRR^PT^ 49 mosquitoes were collected from 12 of the 36 cloth tubes. Of these 80% (*n* = 39) were *An. gambiae* complex, 10% (*n* = 5) were *An. funestus* species group and 8% (*n* = 4) were other anophelines. The DNA of one anopheline female, identified to the *An. gambiae* complex by morphology, did not amplify on multiple attempts (Table 5). Seven of the 16 *An. arabiensis* males (44%) were recaptured marked males. Marked males were only detected until the fourth day of 7 days of collections.

**Table 5:** Summary of anopheline mosquitoes by gender and species sampled from cloth tubes during MRR^PT^, Mamfene, Jozini, KwaZulu-Natal Province, South Africa

Species	Number of males	Number of females	Total (%)
Marked *An. arabiensis*	7	NA	7 (14.3)
Wild *An. arabiensis*	9	21	30 (61.2)
Other *An. gambiae* complex	0	2 (*An. merus*)	2 (4.1)
Total *An. gambiae* complex	16	23	39 (79.6)
*An. funestus* group	1 (*An. rivulorum*)	4 (*An. parensis*)	5 (10.2)
Other anopheline species	1	3	4 (8.2)
Failed amplification	0	1	1 (2.0)
Total collected	18	31	49 (100)

NA, Not applicable (there were no marked *An. arabiensis* females)

Analysis of *An. arabiensis* caught per cloth tube and distance from the release point showed that these were not equally productive in terms of numbers caught (Table 6). Resting site 200.8 was actually the clay pot that is used for routine surveillance which was positioned within 1 m of the location chosen for the cloth tube. The clay pot was, therefore, used instead. It should be noted that mosquitoes collected beyond 100 m from the release point were not used for further analyses as no marked males were recaptured beyond 100 m during MRR^PT^. Marked males (7 in total) were found only in the cloth tubes placed at 50 m (two marked males) and 100 m (5 marked males) (Table 6), making the average dispersal distance of marked males 85.7 m.

**Table 6:** *Anopheles arabiensis* mosquitoes captured per cloth tube per day during MRR^PT^, Mamfene, Jozini, KwaZulu-Natal Province, South Africa

Collection day	Cloth tube number^a^												
	50.1	50.5	100.2	100.4	100.6	100.7	200.5	200.8^b^	200.9	400. 2	400.7	400.12	Totals
Day 1													
Marked ♂	2	–	–	1	–	–	–	–	–	–	–	–	3
Wild ♂	–	–	–	–	–	–	–	3	–	–	–	–	3
Wild ♀	–	–	–	–	–	–	1	3	–	–	–	–	4
Day 2													
Marked ♂	–	–	–	–	2	1	–	–	–	–	–	–	3
Wild ♂	–	1	–	–	–	–	–	1	–	–	–	–	2
Wild ♀	–	1	–	–	–	–	–	2	–	–	–	–	3
Day 3													
Marked ♂	–	–	–	–	–	–	–	–	–	–	–	–	0
Wild ♂	–	–	–	–	–	–	–	–	–	–	–	–	0
Wild ♀	–	–	1	–	–	–	–	–	1	1	–	–	3
Day 4													
Marked ♂	–	–	–	1	–	–	–	–	–	–	–	–	1
Wild ♂	–	–	–	–	–	–	–	1	–	–	–	–	1
Wild ♀	–	–	–	–	–	–	–	1	–	1	1	–	3
Day 5													
Marked ♂	–	–	–	–	–	–	–	–	–	–	–	–	0
Wild ♂	–	–	–	2	–	–	–	–	–	–	–	–	2
Wild ♀	–	–	–	1	–	–	–	–	–	–	–	–	1
Day 6													
Marked ♂	–	–	–	–	–	–	–	–	–	–	–	–	0
Wild ♂	–	–	–	–	–	–	–	1	–	–	–	–	1
Wild ♀	–	–	–	–	–	–	–	2	–	–	–	1	3
Day 7													
Marked ♂	–	–	–	–	–	–	–	–	–	–	–	–	0
Wild ♂	–	–	–	–	–	–	–	–	–	–	–	–	0
Wild ♀	–	1	–	–	–	–	–	–	–	2	–	–	3
Totals													
Marked ♂	2	0	0	2	2	1	0	0	0	0	0	0	7
Wild ♂	0	1	0	2	0	0	0	6	0	0	0	0	9
Wild ♀	0	2	1	1	0	0	1	8	1	4	1	1	20

Only the cloth tubes from which *An. arabiensis* mosquitoes were collected are shown

^a^Cloth tube numbers are given with the first number indicating the distance from the release point and the second number indicating the cloth tube number for that distance. For example, 50.5 is the fifth cloth tube at a distance of 50 m from the release point

^b^200.8 was not a cloth tube but a clay pot. All other resting sites were cloth tubes

#### MRRI

In MRRI, many more mosquitoes were captured at E (*n* = 197) than at C (*n* = 54) (Table 6). *Anopheles arabiensis* made up the majority of the anophelines collected, comprising 64% (*n* = 126) and 79% (*n* = 43) of collections at E and C, respectively. *Anopheles parensis* (of the *An. funestus* group) was the second most abundant species, at 28.5% (*n* = 56) and 9.3% (*n* = 5) at E and C, respectively. *Anopheles quadriannulatus*, *An. merus* and *An. rivulorum* also contributed to the collections (Table 7). Three of 83 (3.6%) *An. arabiensis* males at E and seven of 27 (26%) at C were marked males (Table 8). Marked males were recaptured up to 125 m from release point C (one orange-marked male and one yellow-marked male), but only up to 75 m from release point E (Table 7). The average dispersal distance for released males recaptured from cloth tubes was 70.83 m for males released at C and 58.33 m for males released at E; maximum dispersal distances were 125 and 75 m, respectively. However, one orange-marked male released at E was recaptured at C on day 5 of recaptures. This male had reached a cloth tube located on the 125 m radius from C, approximately 600 m from its original release location (Fig. 4). If this male is included in the average dispersal calculation for males released at E, the average dispersal distance increases to about 194 m.

**Table 7:** Anopheline mosquito species sorted by gender caught from cloth tubes at the central and edge locations during MRRI, Mamfene, Jozini, KwaZulu-Natal Province, South Africa

Species identification and gender	Release point and total captured, *N* (%)	
	E	C
Total * An. arabiensis* males	83 (42.13)	27 (50.00)
Of which were marked *An. arabiensis* males	3 (3.61)	7 [1^a^] (25.92)
*An. arabiensis* females	43 (21.83)	16 (29.63)
*An. parensis* males	33 (16.75)	3 (5.56)
*An. parensis* females	23 (11.68)	2 (3.70)
*An. merus* males	1 (0.51)	0
*An. merus* females	1 (0.51)	2 (3.70)
*An. rivulorum* males	0	0
*An. rivulorum* females	1 (0.51)	2 (3.70)
*An. quadriannulatus* males	1 (0.51)	0
*An. quadriannulatus* females	0	1 (1.85)
No ID male GC	3 (1.52)	0
No ID female GC	1 (0.51)	0
No ID male FG	1 (0.51)	0
No ID female FG	2 (1.02)	1 (1.85)
Escaped female	4 (2.03)	0
Total	197 (100.00)	54 (100.00)

FG,* funestus* group by morphology, GC, *gambiae *complex by morphology; *N*, number of individuals captured,

^a^One male captured at C was an orange-marked male (which was released at E)

**Table 8:** *Anopheles arabiensis* caught per tube distance from each release location during MRRI, Mamfene, Jozini, KwaZulu-Natal Province, South Africa

Distance:	25 m		50 m		75 m		100 m		125 m		150 m		Total	
Location:	C	E	C	E	C	E	C	E	C	E	C	E	C	E
Collection day 1														
Marked	2	–	–	2	1	–	–	–	1	–	–	–	4	2
Wild ♂	2	3	1	1	2	1	–	2	–	2	1	–	6	9
Wild ♀	1	2	–	1	–	1	–	4	–	1	2	1	3	10
Collection day 2														
Marked	–	–	–	–	1	–	–	–	–	–	–	–	1	–
Wild ♂	1	1	1	7	–	3	1	4	–	1	1	4	4	20
Wild ♀	3	2	–	3	–	4	2	3	–	2	–	2	5	15
Collection Day 3														
Marked	–	–	–	–	–	1	1	–	–	–	–	–	1	1
Wild ♂	1	5	1	9	2	2	–	4	–	7	–	3	4	30
Wild ♀	–	–	3	3	1	2	–	2	1	2	–	1	5	10
Collection day 4														
Marked	–	–	–	–	–	–	–	–	–	–	–	–	–	–
Wild ♂	–	–	–	2	–	1	2	1	1	1	1	3	4	8
Wild ♀	–	–	–	1	–	1	–	–	–	–	1	2	1	4
Collection day 5														
Marked	–	–	–	–	–	–	–	–	1^a^	–	–	–	1	–
Wild ♂	2	–	–	3	–	–	–	2	–	4	–	4	2	13
Wild ♀	1	1	1	–	–	–	–	–	–	–	–	1	2	2
Totals													43	124
Marked	2	0	0	2	2	1	1	0	2^a^	0	0	0	7^a^	3
Wild ♂	6	9	3	22	4	7	3	13	1	15	3	14	20	80
Wild ♀	5	5	4	8	1	7	2	9	1	5	3	7	16	41

– = No *An. arabiensis* mosquitoes captured

^a^One marked male was captured at C on day 5 was an orange-marked male that had been released at E

#### MRRII experiment

Sixteen anophelines were captured over five consecutive collection days from six of the 96 cloth tubes (Table 9), of which 50% were *An. arabiensis* (*n* = 8). The remainder of the wild specimens captured were morphologically identified as *An. marshallii* complex with one *An. funestus* group female. Six of the seven (85.72%) *An. arabiensis* males captured were marked males.

**Table 9:** *Anopheles arabiensis* mosquitoes captured in cloth tubes during MRRII, Mamfene, Jozini, KwaZulu-Natal Province, South Africa

Collection day	Trap distance						Total
	25	50	75	100	125	150	
1							
Marked ♂	2	–	2	–	–	–	4
Wild ♂	–	–	–	–	–	–	–
Wild ♀	–	–	–	–	–	–	–
2							
Marked ♂	-	–	1	–	–	–	1
Wild ♂	–	–	–	–	–	–	0
Wild ♀	–	–	–	–	–	–	–
3							
Marked ♂	–	–	–	–	–	1	1
Wild ♂	–	–	–	–	–	–	–
Wild ♀	–	–	1	–	–	–	1
4							
Marked ♂	–	–	–	–	–	–	–
Wild ♂	–	–	–	–	–	–	–
Wild ♀	–	–	–	–	–	–	–
5							
Marked ♂	–	–	–	–	–	–	–
Wild ♂	–	–	–	1	–	–	1
Wild ♀	–	–	–	–	–	–	–
Totals							
Marked ♂	2	–	3	–	–	1	6
Wild ♂	–	–	–	1	–	–	1
Wild ♀	–	–	1	–	–	–	1

### Weather data

The mean daily temperature differed significantly between experiments (ANOVA:* F*_(2, 15)_ = 18.7 *P* < 0.001). Bonferroni post-hoc analysis (critical *T* = 2.694) showed that the mean (± SD) temperature was significantly lower during MRRII (19.20 ± 2.17 °C) than during MRR^PT^ (24.78 ± 2.30 °C) and MRRI (25.38 ± 1.20 °C). The means (± SD) of the daily mean minimum temperatures followed a similar trend, being significantly lower (ANOVA: *F*_(2, 15)_ = 34.8, *P* < 0.001) during MRRII (12.75 ± 2.11 °C) than during MRR^PT^ (19.58 ± 1.87 °C) and MRRI (19.80 ±  0.58 °C) following Bonferroni correction (critical *T* = 2.694). Humidity did not differ significantly between the experiments (ANOVA: *F*_(2, 15)_ = 0.73, *P* > 0.50), with means (± SD) of 71.67 ± 10.61, 74.67 ± 4.27 and 76.33 ± 2.33% RH for MRR^PT^, MRRI and MRRII, respectively. Wind speed was significantly lower during MRRI (mean = 1.08 m/s, SD = 0.098 m/s) than during MRR^PT^ (mean = 2.23 m/s, SD = 0.716 m/s) and MRRII (mean = 2.30 m/s, SD = 0.596 m/s) (ANOVA: *F*_(2, 15)_ = 9.61, *P* = 0.002) after Bonferroni adjustment (critical *T* = 2.694); however, there was no significant difference between MRR^PT^ and MRRII (Additional file 1: Table S2). No rain was recorded during MRR^PT^. During MRRI there was very light precipitation during the morning of the fourth collection day (3 March 2018). During MRRII, rainfall occurred 4 weeks prior to release as well as during the week of the release on the following days: 3 October (4.2 mm), 4 October (8.2 mm), 13 October (4.6 mm), 16 October (6.6 mm), 17 October (5.6 mm), 20 October (3.8 mm) (during the day and evening following the release day). For a summary of the experiments, releases, captures, recaptures and weather data, refer to Additional file 1: Table S1.

## Discussion and conclusion

During this series of MRR experiments the following species were collected from cloth tubes: *An. arabiensis*; *An. merus*; *An. quadriannulatus*; *An. parensis*, *An. rivulorum*; and *An. marshallii complex*. The majority of wild anopheline specimens captured during MRR^PT^ (71.4%) and MRRI (63.4% at E., 76.6% at C) were *An. arabiensis*, while only 20% of wild specimens were *An. arabiensis* in MRRII, with the remainder being *An. marshallii* complex and one *An. funestus* group specimen. Sampling in the same area in 2014 and 2015 confirmed the perennial presence of *An. arabiensis*, with the following species collected: *An. arabiensis*; *An. merus*; *An. quadriannulatus*; *An. parensis*; *An. vaneedeni*; *An. leesoni*; and *An. rivulorum* [4]. These results correspond with the species captured during the present series of MRR experiments, except that no *An. vaneedeni* or *An. leesoni* were detected in our experiments. However, the percentages differed markedly in MRRI at E, where a much higher proportion of *An. parensis* (28.43%) was captured than reported in Dandalo et al. [4] and in the other two MRR experiments. In addition, during MRRII, more *An. marshallii* than any other species were captured. This was the first record of this species since the beginning of the SIT project. The *An. marshallii* complex has been implicated as a potential secondary malaria vector [47] and has become more common in recent field collections. The species diversity we observed is to be expected as the cloth tubes were located outdoors, providing more resting surfaces and possibly being attractive to many different mosquito species. In the experiments performed by Dandalo et al. [4], females were captured more often than males; this was also true in our study for MRR^PT^ but not for MRRI and MRRII. Male-specific traps or resting sites have not been successfully developed yet (but see [48]); however, clay pots and the cloth tubes used in this study have previously been found to be effective for the surveillance of both male and female anophelines [4].

The marked male recapture rates in the present series of MRR experiments were low, ranging from 0.04 to 0.11% for recaptures from cloth tubes. Epopa et al. [27] obtained similarly low recapture rates (0.03–0.08%) using clay pots in Burkina Faso during a series of four MRR experiments. In Epopa et al. [27], clay pots were the least effective method at capturing males (recaptures were performed using three methods: indoor pyrethroid spray catches; clay pots and swarms), with extremely low recapture rates (0.03–0.08%) and these data were excluded from their analyses.

Interestingly, we noted that one clay pot used in place of a cloth tube in MRR^PT^ captured by far the most mosquitoes. This warrants further investigation into resting site or collection device attractiveness. For example: Are clay pots more attractive than cloth tubes? Does the length of time the device has remained in the field for have an effect on collection efficacy? Does it depend on previous use by mosquitoes and the deposition of pheromones that mark the device as a preferred resting site? Is it simply a positional effect? This clay pot had been in place for an extended period of time, having been used for routine surveillance in the area, suggesting that the length of time a trap or resting site has been in the field may have an influence on the number of mosquitoes captured.

The high number of mosquitoes captured at E compared to C during MRRI indicates that the distribution of mosquitoes throughout the target area is heterogeneous. There is possibly a source of mosquitoes near E, in the marshy area between the intended target site and the nearby control site. Depending on resource availability, it may be better to conduct future pilot trials over the area of the intended target site as well as the nearby control site and then use the second control site as the sole control. Alternatively, the target site may be changed to the distant control site. Careful consideration will be required to ensure that the impact of sterile male releases on the population size is measurable. In addition, more estimates, spaced throughout the year, to estimate seasonal population size fluctuations will be beneficial to optimize the timing of releases (as done in [28] and [49]). This is ideally when the population is at its smallest but still receptive to reproduction. Research indicates that reproduction may take place throughout the year for *An. arabiensis* in the study area [50].

The cold conditions (more than 3 °C below the usual October average), along with rainfall during MRRII, probably contributed to the low numbers of mosquitoes captured during MRRII. Paaijmans et al. [51] found that rains caused loss of larvae from water bodies due to the flushing, ejection and killing effects of rainfall. In addition, Charlwood and Edoh [52] suggest that *An. arabiensis* may be more severely affected by rainfall than *An. gambiae*, possibly because *An. arabiensis* immatures are typically found in small temporary larval sites [9, 10] that are prone to desiccation and flooding.

The average wind speed was significantly less during MRRI than during the other two experiments, which may account for the higher swarming activity observed during MRRI than during MRR^PT^ and MRRII. Swarm searches carried out before the MRR experiments were conducted experienced very little success, particularly on windier days. In addition, studies have indicated that flight and swarming activity are negatively affected by wind. Gillies and Wilkes [53] performed upwind tunnel tests using animal bait (CO_2_ and attractive odour source) and found a maximum flight speed for various mosquito species of 1.4–1.8 m/s. At 2 m/s, mosquitoes were no longer captured, instead preferring to settle. Service [54] suggests that wind speeds of ≥ 3 km/h (0.83 m/s) significantly reduce host-seeking flights (female mosquitoes). Munhenga et al. [55] also described a strong correlation between wind speed and mosquito collection in their study, with the proportion of *An. gambiae* complex mosquitoes captured decreasing as wind speed increased. Almost no mosquitoes were collected at wind speeds > 3.1 m/s. At higher wind speeds mosquito flight direction would not be controlled by the mosquito, but it would be subject to the direction of the wind. In this situation, it is likely minimal active dispersal takes place [54]. Therefore, despite the presence of attractive resting sites, the mosquitoes may not be able to direct their flight to select such sites and are, instead, swept along by the wind. This would also likely greatly impact swarming behavior of males. Indeed, in a field cage experiment by Achinko et al. [56], there were no swarms detected on rainy and windy days and females did not leave their resting areas, supporting our interpretation.

In previous work related to the South African SIT project, Munhenga et al. [24] determined that sterilized *An. arabiensis* males needed to be released at a minimum ratio of 3:1 to their wild counterparts to be competitive for females. Using the population sizes calculated from the MRR trials in this study and employing a minimum sterilized to wild male release ratio of 6:1, the numbers required per week for inundation would be approximately 3300 to 37,000 males per hectare using the Lincoln index, which takes daily survival into account [42]. These numbers are based on estimates from MRR^PT^ (November/December) and from MRRI. MRRII was not included in this analysis as no wild males were captured during the first 4 days of collections. The fact that MRRII gave a very low population size estimate may be an indication of how low the population can get during unfavourable periods. Periods when the population is reduced, such as just after winter or before the rainy season in South Africa, may be good times to initiate sterile male releases. Targeting the population after other suppression efforts (such as the application of IRS and larviciding of water bodies) at potential points of low population size will increase the inundative ratio and enhance the likelihood of successful target population suppression using the sterile insect technique.

The average distance travelled by marked males captured in cloth tubes was between 58 and 86 m in this series of experiments. Mean distance travelled, as calculated by Ageep et al. [30] for *An. arabiensis*, was 162 m using males captured from swarms. Mean net dispersal, as determined for *An. coluzzii* by Epopa et al. [27], ranged from 40 to 549 m. Our values fall within the lower ranges reported in these studies. However, mosquitoes are likely able to travel considerably further than the average values obtained, as indicated by the single male recaptured more than 600 m from its release location (and see [57] and [58]). As the cloth tubes in which marked males were recaptured were located at distances of ≤ 150 m from the release points and our recapture rate was low, the dispersal distances are not likely to be representative of actual dispersal, and further studies will be needed to get more accurate results.

The success of the SIT feasibility project hinges on the effective integration of the sterilized laboratory males into the local population in order to compete with wild males for wild females. Laboratory males that can locate and participate in swarms must be at the right place at the right time. During MRRI, we found swarms of wild males that contained 11–50% marked males for swarm sizes of ≥ 2 males. This result is similar to results reported by Ageep et al. [30] who, during their MRR study, recaptured 17.2–42.3% and 26.7–97% marked males from a single large swarm and two small swarms, respectively. Similarly, Epopa et al. [27] captured 76% of marked males from swarms. These results indicate that swarms are probably the ideal locations for obtaining representative samples of males and can be used as traps for population size studies if they are present and accessible.

That laboratory-reared males could locate and participate in swarms with wild males suggests that they have retained natural mating behaviours despite a long colonization period. The colonized strain originated from the area of the study, which may have contributed to their participation in swarms. Marked laboratory-reared males have also been captured in known wild male swarms in Sudan [30, 59]. Epopa et al. [27] used *An. gambiae* (*s.l*) males sourced from the wild, as well as colonized *An. coluzzii* in separate MRR experiments, and found no significant differences in the proportions of recaptures from swarms when these collections were compared. Whether the mating success of laboratory-reared males in the field is equal to that of wild males remains unknown and may be addressed in future experiments. Due to the difficulty in finding swarms in Mamfene, swarms have not been a reliable method for sampling mosquitoes in the region. Several swarms have been observed, but they are not consistently present.

The marking, transport and release of laboratory-reared males from Johannesburg was feasible when performed at the scale reported in this study (5000–15,000 males). Challenges for upscaling the number of mosquitoes needed for the MRR include: sex-separation of mosquitoes, which is laborious and time consuming; marking large numbers of males using a small fridge for knockdown; and transportation to the field site (live adults in BugDorm-1^®^ cages occupy a significant amount of space). Potential ways to mitigate these challenges may include: the development of a sexing system (a genetic sexing strain [22] is available); knockdown in a walk-in cold room for dusting [36] or the use of alternative marking methods, such as Rhodamine B dye [60]; and transport of marked males in compact containers at reduced temperatures [61].

We conclude that the population size estimates obtained in this study can be used as a guide to determine the initial number of males to be used for an SIT pilot trial. Based on the lower population size estimates and a sterile:wild male inundative ratio of 6:1, a minimum of approximately 165,000 sterile males will need to be released per week to cover an area of 50 hectares (3300 sterile males per hectare) in the study site. This may mean that SIT releases should be timed to take place just before the rainy season (before the population size begins to increase), or following a drive to suppress the target population using other methods, such as after IRS campaigns. Due to the heterogeneous nature of the study site and the close proximity to a potential larval site, adjustment of the target site selected may be required prior to conducting SIT pilot trials.

## Supplementary Information

**Additional file 1: Table S1.** Mamfene, KwaZulu-Natal MRR experiments summary information. **Table S2.** Wind direction and speed averages for the pilot trial (MRR^PT^), MRRI and MRRII.

**Additional file 2.** Sweep net design.** A** Sweep net. Net length: ~ 1.5 m, hoop diameter: ~ 70 cm, handle: ~ 2 m.** B** Mid notch and lashing.** C** Top notch and lashing.

## Data Availability

The dataset generated by this study is available from the corresponding author upon reasonable request.

## Abbreviations

C: Central release area
E: Edge release area
IRS: Indoor residual spraying
KZN: KwaZulu-Natal
MRR: Mark–release–recapture
RH: Relative humidity
SD: Standard deviation
SIT: Sterile insect technique
