# Supplementary figures and images for: Estimates of the population size and dispersal range of Anopheles arabiensis in Northern KwaZulu-Natal, South Africa: implications for a planned pilot programme to release sterile male mosquitoes
Source: Parasit Vectors. 2021 Apr 19;14:205. doi: 10.1186/s13071-021-04674-w (PMC8056555; doi:10.1186/s13071-021-04674-w)

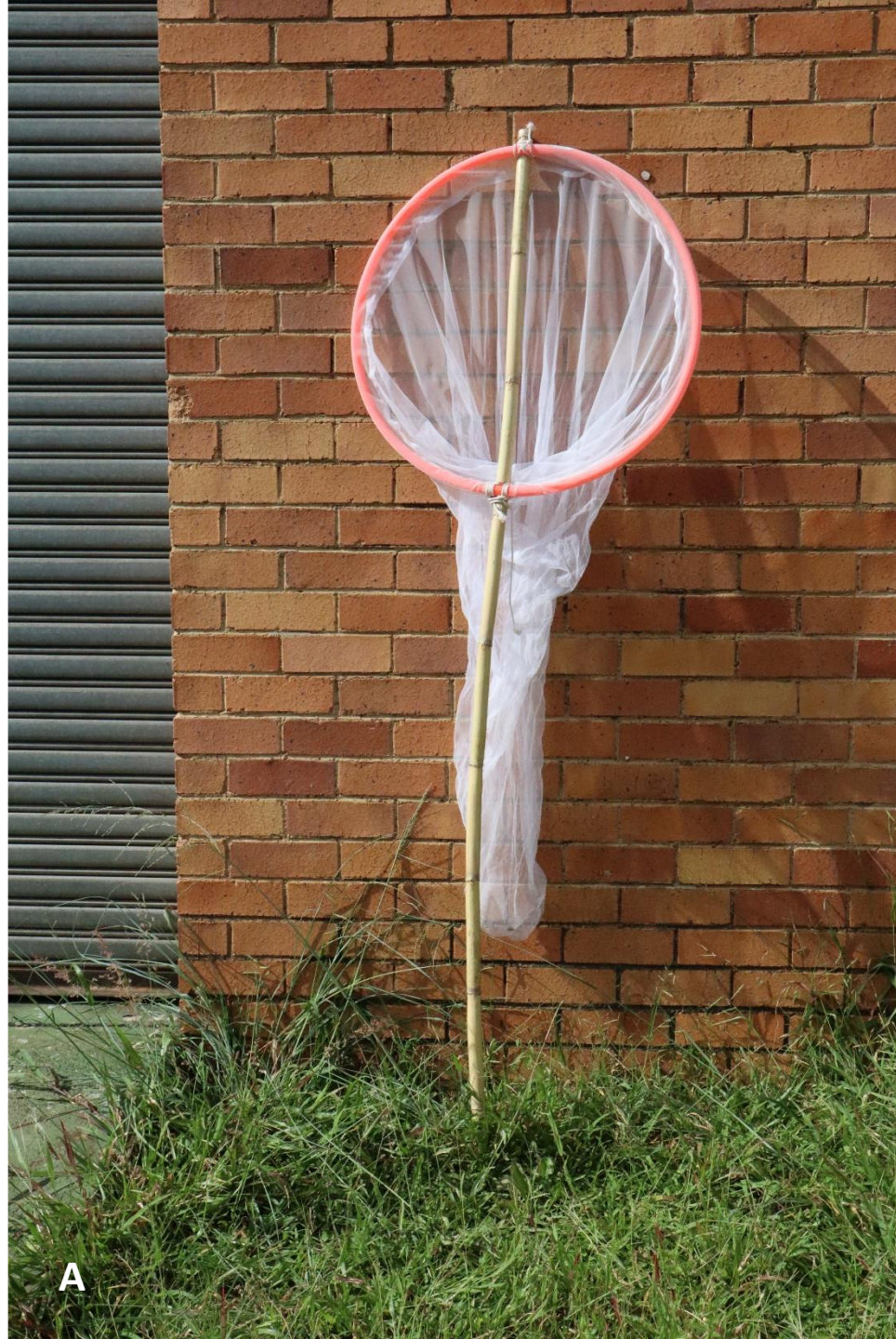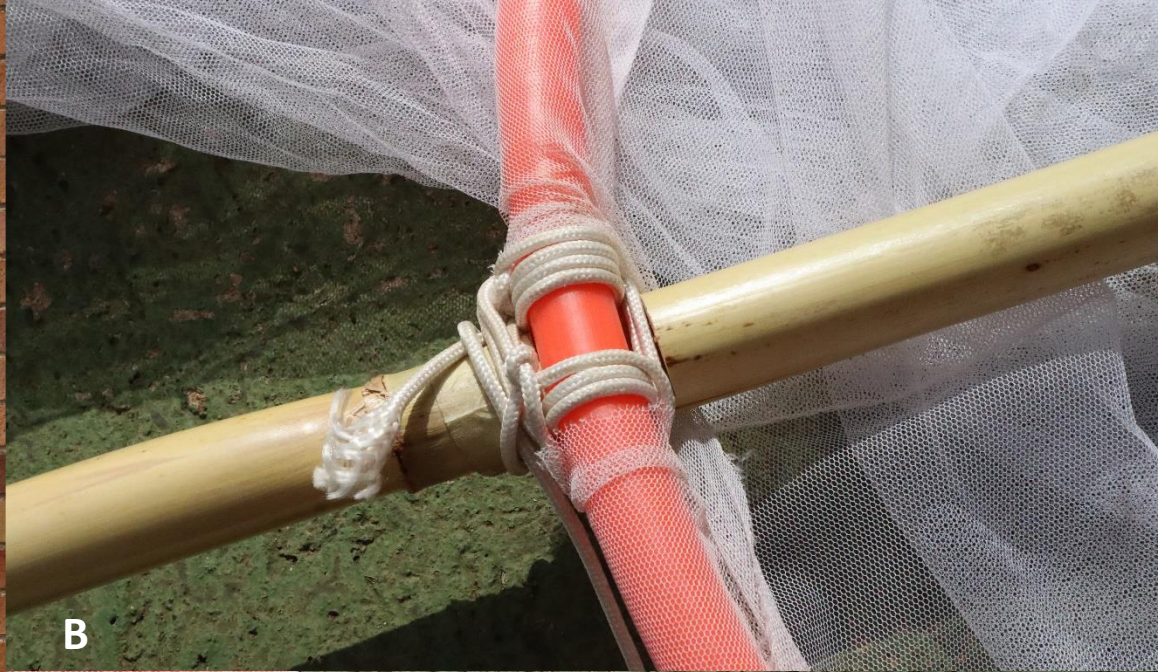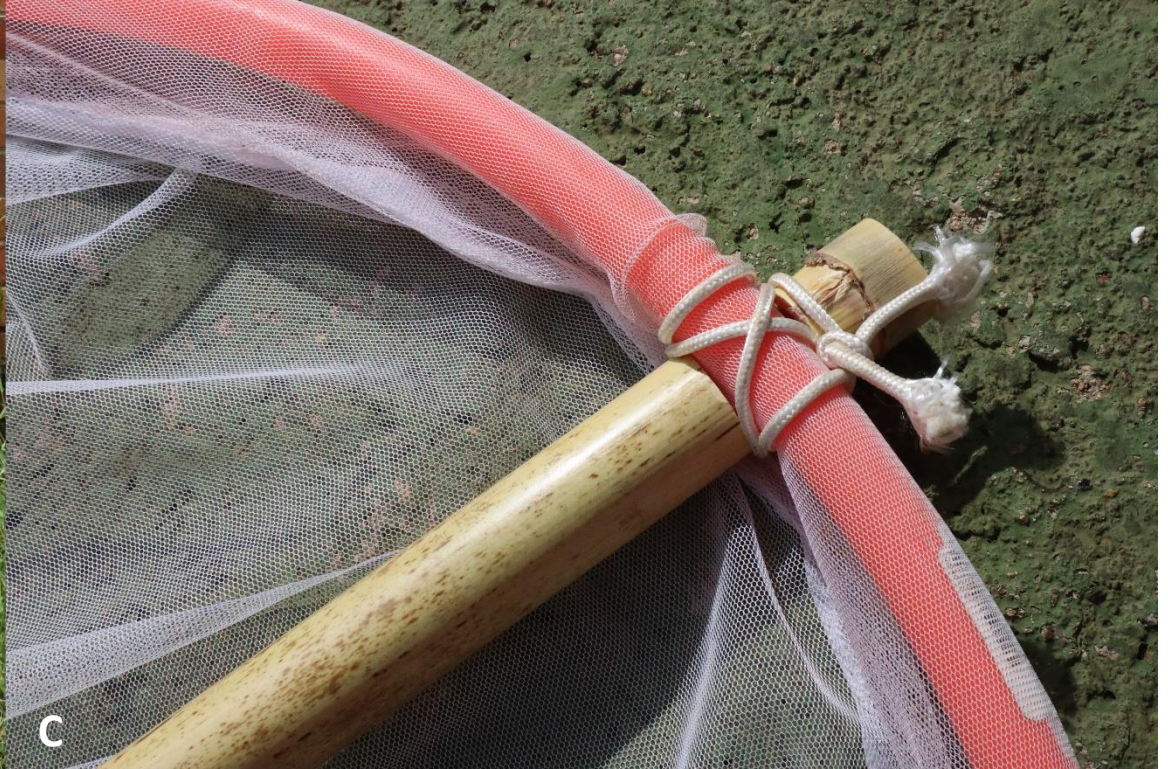

Supplement: Supplementary file 2 — Additional file 2. Sweep net design. A Sweep net. Net length: ~ 1.5 m, hoop diameter: ~ 70 cm, handle: ~ 2 m. B Mid notch and lashing. C Top notch and lashing. [file 13071_2021_4674_MOESM2_ESM.pdf]
